# Addressing Cardiovascular Health Disparities in Minnesota: Establishment of a Community Steering Committee by FAITH! (Fostering African-American Improvement in Total Health)

**DOI:** 10.3390/ijerph16214144

**Published:** 2019-10-28

**Authors:** Chandrika Manjunath, Oluwatomilona Ifelayo, Clarence Jones, Monisha Washington, Stanton Shanedling, Johnnie Williams, Christi A. Patten, Lisa A. Cooper, LaPrincess C. Brewer

**Affiliations:** 1 Department of Cardiovascular Medicine, Mayo Clinic College of Medicine, Rochester, MN 55905, USA; manjunath.chandrika@gmail.com; 2 Mayo Clinic Alix School of Medicine, Rochester, MN 55905, USA; Ifelayo.Oluwatomilona@mayo.edu; 3 Hue-MAN Partnership, Minneapolis, MN 55409, USA; bobcjones@hotmail.com; 4 Volunteers of America, Minneapolis, MN 55439, USA; monishawash@gmail.com; 5 Cardiovascular Health Unit, Minnesota Department of Health, St. Paul, MN 55164, USA; stanton.shanedling@state.mn.us; 6 Full Proof Ministry Church of God in Christ, Crystal, MN 55429, USA; jbw1107@gmail.com; 7 Department of Psychiatry and Psychology, Mayo Clinic College of Medicine, Rochester, MN 55905, USA; patten.christi@mayo.edu; 8 Division of General Internal Medicine, Department of Medicine, Johns Hopkins University School of Medicine, Baltimore, MD 21205, USA; lisa.cooper@jhmi.edu

**Keywords:** African-Americans, cardiovascular health, community-based participatory research, community-engaged research, community steering committee, health disparities, health equity

## Abstract

Despite its rank as the fourth healthiest state in the United States, Minnesota has clear cardiovascular disease disparities between African-Americans and whites. Culturally-tailored interventions implemented using community-based participatory research (CBPR) principles have been vital to improving health and wellness among African-Americans. This paper delineates the establishment, impact, and lessons learned from the formation of a community steering committee (CSC) to guide the Fostering African-American Improvement in Total Health (FAITH!) Program, a CBPR cardiovascular health promotion initiative among African-Americans in Minnesota. The theory-informed CSC implementation process included three phases: (1) Membership Formation and Recruitment, (2) Engagement, and (3) Covenant Development and Empowerment. The CSC is comprised of ten diverse community members guided by mutually agreed upon bylaws in their commitment to FAITH!. Overall, members considered the CSC implementation process effective and productive. A CBPR conceptual model provided an outline of proximal and distal goals for the CSC and FAITH!. The CSC implementation process yielded four lessons learned: (1) Have clarity of purpose and vision, (2) cultivate group cohesion, (3) employ consistent review of CBPR tenets, and (4) expect the unexpected. A robust CSC was established and was instrumental to the success and impact of FAITH! within African-American communities in Minnesota.

## 1. Introduction

Despite improvements in the general population, African-Americans (AAs) continue to bear the greatest burden of cardiovascular disease (CVD) incidence and mortality, particularly compared to whites [1,2]. Despite being ranked the fourth healthiest state in the United States, Minnesota (MN) is not immune to these disparities [3]. MN AAs have higher CVD incidence and nearly double the mortality rate from CVD than whites [4]. Efforts to mitigate these inequities are further complicated by discrimination at systemic levels and legacies of historic mistreatment of AAs [2]. Compared to other racial/ethnic groups, AAs have poorer socioeconomic and education levels, and associated excess adverse outcomes contributing to persistent health inequities [2]. This is exacerbated by the aversion of many AAs to health-related research participation, owing to past exploitation in clinical investigations like the Tuskegee Syphilis Study [5]. Consequently, AAs lack trust and involvement in research that could ultimately benefit them.

Community-based participatory research (CBPR) has demonstrated potential as a means to renew the confidence of AAs in research participation as it fosters trustworthy and productive relationships between researchers and communities [6]. This approach places emphasis on community member inclusion throughout the entire research process as valuable partners rather than as research subjects. Many CBPR-driven projects focus on reducing health disparities through community-level initiatives [7] in non-traditional community settings—such as AA churches and barbershops [1,8]. Some such partnerships also include community steering committees (CSCs) or community advisory boards (CABs). These are associations of multidisciplinary community representatives who provide nuanced insights on local contexts, partake in the research process alongside researchers, and disseminate project information to their communities and beyond [6,9]. To this end, Mayo Clinic investigators with the shared vision of organizational and community stakeholders, developed Fostering African-American Improvement in Total Health (FAITH!), a CBPR program focused on cardiovascular health (CVH) and wellness promotion for MN AAs through a novel, culturally-tailored mobile health (mHealth) intervention [1,10].

FAITH! was initially born out of a class assignment during the public health studies of the project principal investigator (PI, L.B.) in 2008 in Baltimore, MD. As a charge to create a potentially sustainable, community-based intervention addressing chronic disease disparities among racial/ethnic minority groups, her group developed a nutrition education program in partnership with a local AA church using a CBPR approach [11,12]. The program resulted in healthy lifestyle changes among the participants and was of great benefit to the congregation as a whole. Upon moving to Rochester (RST), MN, the PI built relationships with additional local AA churches that were privy to her work in Baltimore and were keen on expanding FAITH! to their congregants [13]. In 2013, a formal CBPR partnership was solidified with the churches and the program redirected its focus to CVD prevention based on the expressed needs of the community. Given the significant impact of FAITH! [1] on the CVH of AAs in RST and expressed desires of FAITH! participants to extend the reach of the program, a study-specific CSC was established to increase its accessibility to other MN AA communities [10]. The PI and church partners jointly agreed upon expanding to Minneapolis-St. Paul (MSP), as this area has the largest proportion of AAs in MN with clear CVH disparities [14]. A goal was set to forge a new partnership framework between the academic research team and the MSP AA community through a CSC comprised of diverse community leaders from RST and MSP. The CSC would provide infrastructure for long-term sustainability of FAITH! in confronting CVH disparities in MN.

This article describes: (1) the process of creating the FAITH! CSC and bylaws, (2) the significant impact of the CSC on the FAITH! Program, and (3) lessons learned. The implementation process and lessons learned could provide a framework for other investigators using a CBPR approach to promote health equity in underserved communities.

## 2. Methods 

First, background is provided on CSC conceptualization. Next, the CSC objectives are described. Finally, CSC implementation is outlined, which consisted of three phases: (1) Membership Formation and Recruitment, (2) Engagement, and (3) Covenant Development and Empowerment.

### 2.1. Academic-Community Partnership Development: First Steps 

The central FAITH! academic-community co-leader study team is comprised of the FAITH! PI, a cardiologist from Mayo Clinic in RST, MN (L.B.), and a former director of a federally qualified health center and community advocate in Minneapolis, MN (C.J.). They were connected in May 2015 through the director of the MN Department of Health Cardiovascular Health Unit in St. Paul, MN (S.S.), in light of their shared interest in CVD prevention in underserved communities. One initiative led by the community partner (C.J.) was “Clippers ‘N Curls,” a community-level outreach program that mobilized AA barbershops and salons to reduce CVD and stroke incidence in MSP AA communities [15]. The program equipped barbers and hair stylists with CVH promotion resources including educational videos to share with clients as well as blood pressure monitors to screen clients for hypertension [15,16].

The co-leaders met again shortly after their initial meeting and agreed upon forming an academic-community CBPR partnership between the medical institution and faith-based and community organizations. The partnership would focus on CVH promotion among AAs by engaging AA churches in RST and MSP areas. Seeking to enrich their partnership and CBPR knowledge, the co-leaders were one of 12 academic-community teams selected through a competitive review process to attend the Detroit Community-Academic Urban Research Center (DURC) CBPR Partnership Academy in July 2016 [17]. It provided a rich co-learning forum to partake in formal coursework, experiential CBPR learning, professional development sessions, and field experiences to engage with local community groups and members of the DURC CAB “in action” [17]. The Academy provided the co-leaders the impetus to form a CSC, which they viewed as a means to provide infrastructure for FAITH! and engage with community representatives. While both CABs and CSCs involve community members to receive feedback on research projects, the co-leaders agreed that the term CSC was most reflective of the central purpose of the group to “steer” FAITH! forward in addressing the needs and preferences of the community. The co-leaders secured a DURC seed grant, which supported CSC creation and participated in a year-long mentorship program with a CBPR Partnership Academy-affiliated academic researcher and community partners. Throughout the year, DURC-led learning activities including online webinars, and mentoring calls were hosted. Teams also gained access to a national CBPR Partnership Academy Network of Scholars to sustain discussion and idea sharing among participants [17]. Considering the novelty of FAITH! in MN AA communities with little existing frameworks of community-wide advisory groups, the DURC resources were invaluable to providing the co-leaders directionality in creating a CSC.

From antecedent headway with FAITH!, the study PI had existing relationships with RST churches and church-designated liaisons keen on extending the research partnership to MSP [1]. The process of broadening program efforts relied heavily on community trust-building alongside the community-partner and his established relationships in MSP [18].

### 2.2. FAITH! Community Steering Committee Objectives

**Figure 1:**
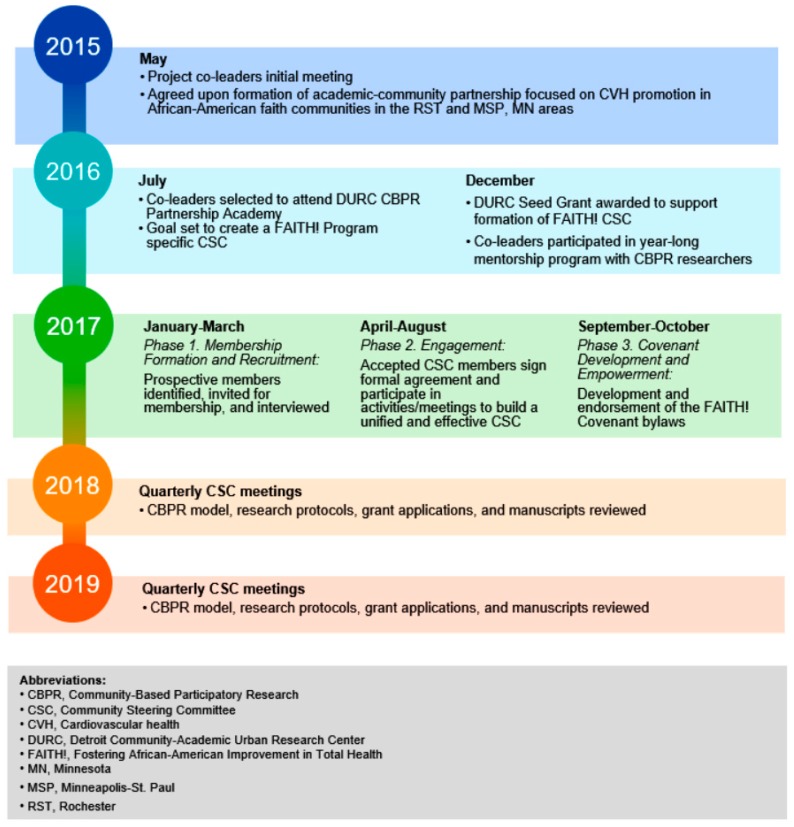
Timeline of FAITH! Community Steering Committee Formation and Implementation.

CSC creation (Figure 1) took place within the broader FAITH! CBPR study. The co-leaders strategically-planned CSC formation informed by the social cognitive theory [19] and community-building and mobilization model [20]. It was imperative to have a CSC and intervention built upon sound theoretical frameworks to increase its widespread applicability if proven effective. The social cognitive theory describes the interplay and influence of individual experiences, actions of others, and environmental factors on self-efficacy [11,19]. Contextualized in the CSC, efficacy defined as the belief of the collective in its capability to achieve a purpose [21]. Internalizing this among CSC members involved prioritizing their distinct skills, creating an environment of transparency and support, and disseminating outcomes of their efforts to communities and beyond. The community-building and mobilization model utilizes the constructs of building community members’ awareness of their collective abilities, guiding them in identifying key health issues and strategies to tackle them, and enlightening them of their “shared power, resources, and ability to set their own agenda” [11,20]. 

To accomplish such a CSC, the following objectives were outlined:

1. Establish a CSC specific to FAITH! composed of representatives from local AA faith- and community-based organizations to provide infrastructure for community members to voice concerns, priorities, and preferences in the development of a culturally appropriate research process and intervention.
2. Define a process for the formation, operation, and maintenance of the academic-community partnership and CSC using a CBPR approach through the development of a FAITH! Covenant.

### 2.3. Community Steering Committee Implementation Process: Three Phases

The co-leaders planned structured activities to accomplish CSC objectives in three phases: 

•  Phase 1. Membership Formation and Recruitment (January–March 2017)

The co-leaders devised a formalized process to determine CSC membership composition and recruitment strategies [18,22]. They brainstormed prospective members representing reputable local organizations, emphasizing formation of a diverse group to position the project favorably within the AA community. They focused on the AA community as a “unit of identity,” defined as a sense of shared values and goals, such that the CSC would build upon community strengths and resources [23,24]. Selection criteria were proposed utilizing a “potential member matrix,” carefully considering the following: reputation, activities and achievements in the community, CVH expertise, and interest in community health and advocacy [18]. A goal was set to recruit 10 members representing diverse stakeholders. Through online searches and established community networks, potential members from RST and MSP were identified. Invitations for membership were emailed and in-person or telephone interviews were held with those responding with interest. Both co-leaders were present for all interviews during which potential roles, responsibilities, and clarification of the CBPR partnership expectations were reviewed. Recruited members signed a formal letter of mutual intent (Appendix A) as a shared understanding of their role in the CSC and as a means of minimizing possible misunderstandings. They were made aware of the time commitment involved, including monthly to quarterly (every 3 months) meetings each of approximately two hours in duration. It was also relayed that a small honorarium would be offered to CSC members as appreciation for their time and that meals would be provided at all meetings. All but one individual accepted CSC membership. This individual suggested another community member in their place. However, the co-leaders respectfully explained the recruitment procedure they employed to ensure a well-positioned CSC and thus invited an alternate member through this process. The recruited CSC members were representatives of a diverse conglomerate of faith, government, and community-based organizations, health systems, and national/regional associations (Table 1). 

•  Phase 2. Engagement (April–August 2017) 

Two meetings were held towards building a cohesive and effective CSC. The co-leaders were designated co-chairs of CSC meetings. In March 2017, they also attended a Johns Hopkins University Center for Health Equity CAB meeting, which provided the co-leaders an exemplar for the CSC as it progressed. In addition, the co-leaders received advice from their assigned CBPR Partnership Academy mentors to first invite all CSC members to meet each other in a low-key environment for social networking. Thus, the co-leaders planned a retreat to accomplish this goal. 

1.  Team-building retreat (May 2017) 

The primary aim of the retreat was to promote long-term commitment to sustainability and encourage members to appreciate their collective strengths [23]. The three-hour retreat (Appendix B) was held at a local community center, allowing members to engage with one another in an informal and accessible environment, and included interactive team-building and physical activities (e.g., Zumba) to shape an internal culture of health and wellness. The co-leaders provided materials on overall FAITH! and CSC goals, CBPR tenets with case studies, co-leader media coverage and publications, and biographies of members and their respective organizations.

2.  Operating Procedures and Maintenance Meeting (August 2017) 

This meeting concentrated on building team dynamics and defining CSC functioning. Members discussed their research participation perceptions and experiences, and group meeting best practices to guide CSC communication and decision-making. The group decided upon a ‘70% consensus rule’ as a means of reaching agreement on low stakes decisions (e.g., scheduling meetings) in which 70% of members are required to agree for consensus [18]. It was decided to dedicate portions of meetings for members to provide feedback on study design, implementation, and dissemination. This information would be presented using lay terms and minimal medical jargon to ensure all members of our multidisciplinary group could interpret and analyze the information. Utilizing a variety of presentation formats was also preferred to appeal to all learners including visuals through presentation slides, handouts, videos, and infographics. Specific time allotted for open discussion during meetings was also recommended. Members were presented with several options to gauge their satisfaction and for process evaluation of CSC effectiveness, including qualitative (e.g., key informant interviews) and quantitative (e.g., surveys) methods, and collectively decided upon surveys as the preferred means. Furthermore, members defined their meaning of project sustainability to assist the co-leaders in reinforcing which CBPR criteria to integrate into study components. Following this meeting, the co-leaders summarized its proceedings for future discussion.

•  Phase 3. Covenant Development and Empowerment (September–October 2017)

Three sequential meetings were held to mutually develop, review, and endorse the CSC bylaws, which would aim to empower the CSC towards accomplishing a unified vision, mission, and purpose.

1.  Meeting 1. Covenant Development (September 2017) 

During Meeting 1, as summarized from prior meeting proceedings, members discussed CSC operating norms, process evaluations, and sustainability plans and from this delineated bylaws as a set of guiding principles to cultivate a joint and action-oriented CBPR project [9]. Adapted from Udoh et al., the CSC embraced the name “Covenant” to encompass the bylaws, given its association with faith and spirituality. Defined as a “formal, solemn, and binding agreement for the performance of some action” used to usher a CBPR project, the Covenant defined CSC aims and aligned with the AA faith community [9]. An incipient Covenant was drafted by a CSC subcommittee and distributed through email for iterative review by members. The co-leaders incorporated feedback into the draft and circulated it to members for forthcoming discussion.

2.  Meeting 2. Covenant Review (October 2017)

Given the considerable constructive feedback provided by the CSC from Meeting 1 and email correspondence, this ad hoc meeting was held to comprehensively review the comments collectively. This additional input was consolidated by the co-leaders into the revised Covenant. 

3.  Meeting 3. Covenant Endorsement (October 2017) 

**Figure 2:**
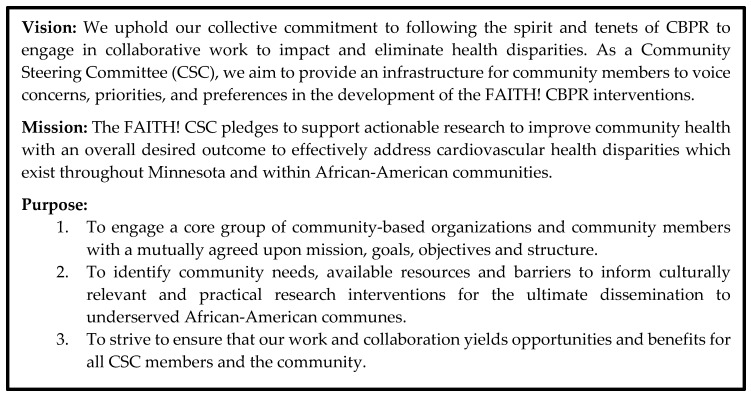
FAITH! Community Steering Committee Covenant: Vision, Mission, and Purpose.

Final Covenant modifications were made, followed by a unanimous vote of its endorsement. The Covenant (Figure 2) would serve as a “living document” to review for accountability to FAITH!, and also inspiration and empowerment towards improving CVH in the AA community. It contained bylaws outlining membership, governing structure, subcommittees, voting procedures, meeting frequency and structure, CSC support staff, and budget management. For instance, the CSC decided upon having a minimum of 10 and maximum of 15 members, expected to serve at least a one-year term, attend 50% of quarterly meetings in-person or by teleconference, and be actively involved in and prepared for CSC programming. The group resolved that project findings be shared in layman’s terms to ensure equitable member understanding and involvement in the research process. To approve high stakes decisions (e.g., Covenant amendments), a majority vote of 6/10 members’ approval was defined. The Covenant was to be reviewed at least annually, with amendments requiring a majority vote for passage and inclusion. Finally, meetings were to be held at convenient community sites, opened with prayer, followed by approval of past meeting minutes and member updates on FAITH!-related and community activities.

### 2.4. FAITH! and Community Steering Committee Conceptual Framework 

**Figure 3:**
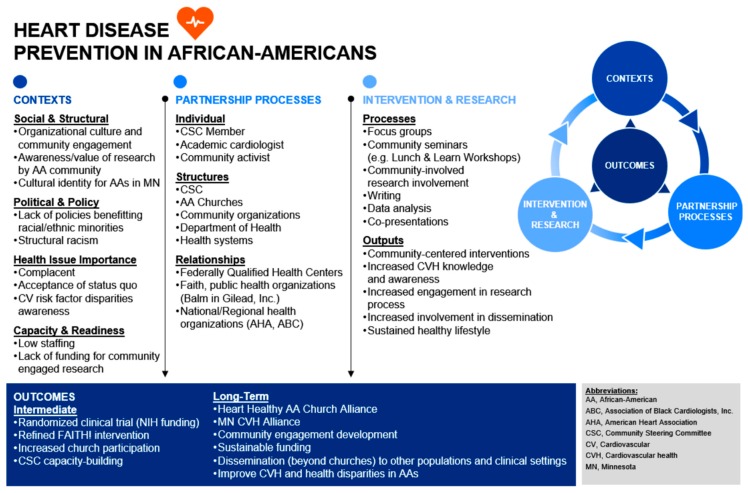
Community-based participatory research (CBPR) conceptual model for the FAITH! Community Steering Committee.

The CSC encouraged an environment for FAITH! to grow and further involve community members in this progress, better honor CBPR doctrines, and translate theory into practice [25]. This was in part due to a unifying model, which are crucial to projects involving diverse stakeholders [26]. The CSC was actively involved in constructing a CBPR model, a conceptual framework tool utilized as a malleable blueprint to frame proximal and distal goals for the overarching FAITH! Program (Figure 3) [25,26,27]. It provided a fluid dynamic schema to display interconnections between outlined components (Contexts, Partnership Processes, Intervention and Research, Outcomes) anticipated to evolve as FAITH! progresses [27]. The model allowed for tangible holistic visualization of how each domain and its linked elements collaborate to yield the utmost benefit for the prioritized community. It enabled the CSC to plan and evaluate contexts (e.g., sociopolitical), stratify partnership processes (e.g., individual/structural), and review intervention and research project components (e.g., processes/outputs) while focusing on intermediate and long-term outcomes [26]. Over the year following model creation, it was collectively reviewed and revised as necessary.

## 3. Results

### 3.1. Community Steering Committee Member Evaluations 

Members completed post-meeting evaluations for Phases 2–3 to gauge CSC formation process effectiveness, whether expectations were met, and views on meeting productivity. Evaluation feedback is summarized in Table 2. Overall, feedback was largely positive, with nearly all participants (95.2%) affirming that meetings were worth their time, that meeting objectives were accomplished (91.6%), and that meetings allowed them to contribute and share in community partnership development (96.0%). They supported the action plans and strategies established by the CSC, with the majority viewing them as good or excellent (78.3%). All members indicated high ratings for the outline of next steps/follow-up for the CSC and FAITH!. In 2018, members also completed an annual review evaluating the CSC overall (Table 3). There was a 70% completion rate of the annual review survey. Results were similarly positive with all respondents reporting that they agreed or strongly agreed that the CSC is fulfilling their expectations and has a clear mission/vision. All members strongly agreed that the CSC is focused on improving community health. Open-ended questions gleaned constructive feedback on suggested potential CSC members, amendments to the FAITH! Covenant, and expressions of content with the overall structure and functioning of the CSC. 

### 3.2. Community Steering Committee Accomplishments

Our CSC implementation process provided the infrastructure for the success of CSC formation. The CSC continues to thrive through ongoing productive quarterly meetings, research projects, and community outreach. Feedback from the member evaluations was used to further amend the FAITH! Covenant with a focus on goal-setting with timelines. Input on CSC aspects that should be continued was applied to ongoing quarterly meeting formatting as well as community engagement and outreach event planning. A FAITH!-specific quarterly newsletter was identified as a means to display project progress and accomplishments and how it is actively addressing CVH in the AA community. This information was disseminated to CSC members and community partners. 

**Figure 4:**
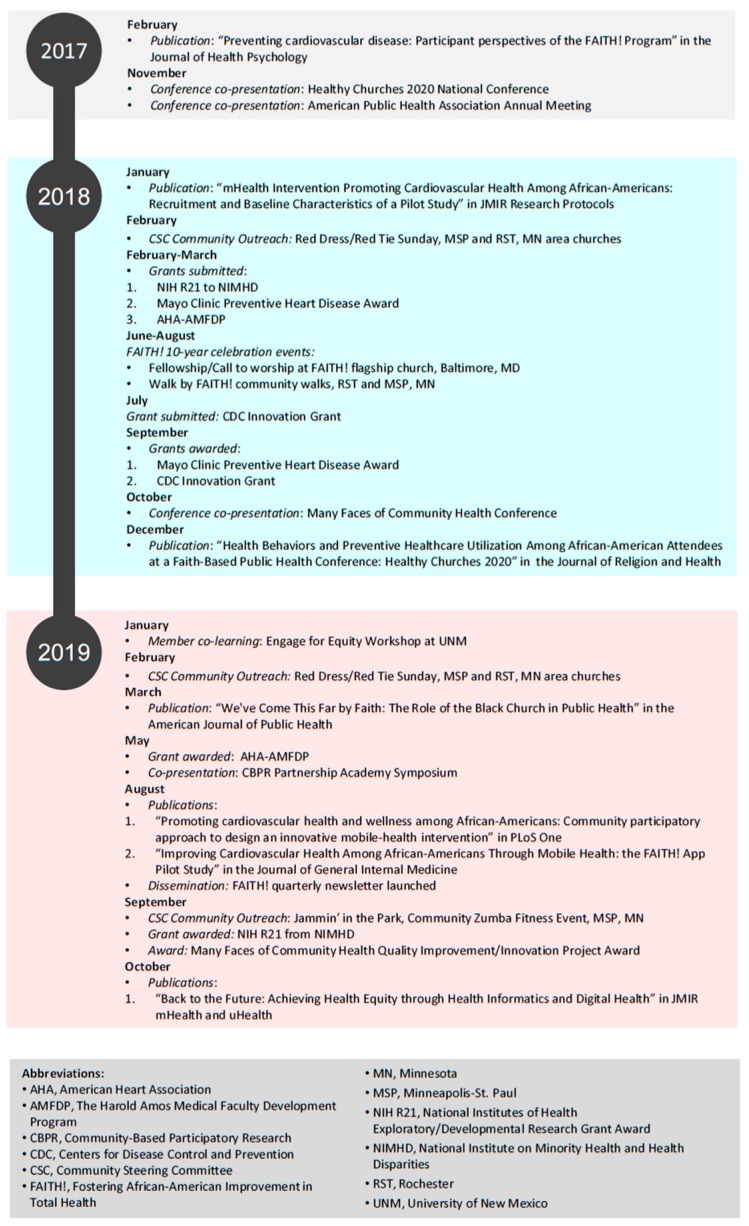
FAITH! Community Steering Committee Activities and Accomplishments.

Many activities and accomplishments have resulted from utilization of the guiding CBPR model (Figure 4). Alongside the co-leaders, CSC members have reviewed study protocols, co-authored manuscripts [10,13,28,29,30,31], and been involved in applying for, reviewing, and securing grants [10,32,33,34]. Members have also been heavily involved with FAITH! Program dissemination efforts and have co-presented at regional and national conferences [17,35,36,37]. In January 2019, the co-leaders and a CSC member (M.W.) were invited to attend the Engage for Equity Partnership Evaluation Workshop, a joint learning session with CBPR partnerships from across the country [38]. Members have planned and participated in community events such as the Walk by FAITH! in celebration of the ten-year anniversary of the founding of FAITH! in Baltimore, which had great success reaching over 400 community members [39]. In honor of American Heart Month, the CSC has held commemorative events, including Red Dress/Red Tie Sundays at local AA churches. In May 2019, the co-leaders and a CSC member (M.W.), attended the CBPR Partnership Academy Symposium, “Promoting CBPR to Achieve Health Equity”, which featured participants in the CBPR Partnership Academy Network of Scholars. They presented updates on the FAITH! Program including the CSC and its challenges, lessons learned, and successes. These activities reinforce the value of a CSC in empowering the evolution of FAITH! and its impact on CVH promotion in the AA community.

## 4. Discussion

This paper describes the development of a CSC from the formation of a central academic-community partnership into a multidisciplinary group of stakeholders to further strengthen and support the FAITH! Program. The FAITH! Covenant, as mutually agreed upon bylaws, cultivated productive academic–community team interactions and enhanced overall project objectives and sociocultural relevance. Multiple Covenant iterations supported group cohesion by creating a document most accurately encompassing the beliefs and input of all members. Furthermore, the CSC extended FAITH! further credibility within the local AA community given its inclusion of trusted and highly regarded community partners. Committing to a CBPR approach facilitated CSC formation and required flexibility, patience, and relationship-building. Reviewing CBPR principles early in CSC formation was underscored at the CBPR Partnership Academy attended by the project co-leaders. Continued emphasis on these principles throughout each implementation phase nurtured trust between the co-leaders and CSC members. The dedication of members to the CSC, FAITH!, and community outreach has bolstered the proliferation of FAITH! and its mission to promote a CVH intervention among AAs with possible far-reaching applicability.

### 4.1. Lessons Learned in Forming a Community Steering Committee

FAITH! was the first CBPR partnership focused on CVH in AAs in MN, thus the co-leaders had scant existing bodies of work to follow or build upon. Also, MN is comprised of a predominantly white population, which oftentimes presents a challenge of marginalization to AAs within the state [13]. While there are a number of CBPR studies also involving CABs/CSCs among AA communities outside MN, the large majority focus on AAs in states with higher proportions of AAs compared to MN [5,8,40,41]. These studies demonstrate the importance of CBPR for engaging AAs in the research process, particularly because of historical mistrust and marginalization [5,10]. CBPR and CABs/CSCs enable community members to voice concerns and advise on appropriate and respectful interventions for the AA community and its distinct sectors. There have been a number of CBPR studies prioritizing AAs by engaging faith-based organizations as religiosity/spirituality and the Black church are valued by many AAs [29,42]. Our study and CSC build upon and expand these studies by forging frontiers in an understudied and underserved group of AAs through community engagement. We comprehensively describe the methodology of forming, implementing and evaluating our CSC, which fulfills a major gap in the CBPR literature. The process of constructing a CSC from the ground up resulted in the following lessons learned by the co-leaders. 

#### 4.1.1. Clarity of Purpose and Vision Is Essential 

The co-leaders made conscientious efforts to ensure all members had a clear understanding of CSC expectations, purpose, and vision from the outset. Resulting from ensuring an unambiguous grounded space for the CSC, this clarity led to meeting deliverables according to the desired timeline despite transitory obstacles. All CSC members were content with the timeline and accomplishments of each CSC implementation phase. Having distinct aims with anticipated timelines for each phase allowed for the CSC to remain on one accord and goal-oriented. The academic partner learned specific strategies from CSC members on how to more clearly communicate the overall project purpose in the context of the local AA community needs, which allowed for the CSC to better formulate its vision to support the fulfillment of the project purpose. Investment and transparency in all CSC dimensions engendered a sense of responsibility among the team as a whole to join forces in eliminating CVH disparities within the AA community. 

#### 4.1.2. Group Cohesion Must Be Cultivated 

The retreat inspired group harmony and a collective spirit towards improving community health. Cohesion was also supported by commencing each CSC meeting with member updates to learn from and distinguish the merit of their unique attributes. Additionally, discussing individual contrasting ideas contributed to fostering critical thinking and team-building constructive forces in project development [43]. The CSC illustrated the significance of reinforcing CBPR principles throughout the research process as it enhanced group trust and consensus, which has been vital to the accomplishments and productivity of FAITH!. Finally, the Covenant enhanced trust by way of being a mutually agreed upon vision and living document, connecting members to one another and FAITH!. 

#### 4.1.3. Employ Consistent Review of CBPR Tenets with the CSC

Many CSC members were accustomed to “top down” versus “lateral” approaches to collaboration. That is, being sounding boards rather than equally salient. During every meeting, the co-leaders acknowledged and parsed CBPR tenets to underscore the equitable nature of all member input and feedback. This process also supported mutual re-evaluation of the tenets to identify if and how they could be re-applied as FAITH! evolved. As an embodiment of the co-partnering nature of CBPR, the CSC increased the capacity of FAITH! to fulfill its overarching goal to benefit the AA community. 

#### 4.1.4. Expect the Unexpected 

The intended timeline for Covenant creation was prolonged, as members did not reach consensus at the anticipated pace. A need to alter the timeline was promptly identified and accordingly an additional meeting convened without hindering progress, while acknowledging that this deviation only displayed the CSC members’ diligence and dedication. Moreover, when an alternate CSC member was suggested by an interviewed community member who declined, the member selection matrix protocol was utilized to strategically navigate this potentially delicate situation while providing both individuals with warranted respect.

### 4.2. Value of a Community Steering Committee

When creating FAITH!, there were minimal pre-existing structures on which to model a CBPR project and a lack of working relationships between the academic institution and the local AA community. Nonetheless, having space to mindfully construct a framework allowed for conceptualizing the most community-specific program alongside community members, and subsequently the CSC. While our CSC shares characteristics with those of other CBPR studies, it is unique in that it arose from the expressed desire of study community partners and participants themselves as a means to effectively increase the reach of the overarching program to other underserved communities. In this sense, they were co-creators with the study team. Nonetheless, CABs and CSCs have been effective whether formed prior to or following initiation of research projects [18,44,45,46,47]. Our CSC formation and implementation process further validates the clear benefits of investing time in capacity-building for sustainable partnerships between academic institutions and underrepresented communities in research to address health disparities. It also demonstrates that these marginalized groups, including AAs, are keen on integrating themselves into the research process through culturally sensitive means such as CABs/CSCs [47]. Our outline of achievements of our CSC are not merely for exhibition, but to demonstrate to other academic–community partnerships what can be accomplished through the establishment of a goal-directed CSC.

It has been consistently demonstrated that transparency in CBPR is requisite to the emergence and maintenance of mutual trust between partners [5,6]. Other CBPR investigators have stressed an “open door policy” for communication within groups. This method enables capacity building and bridging gaps between researchers and community members by inspiring member engagement as they see their viewpoints are valued and integrated regardless of their positioning [48]. Akin to the FAITH! CSC, it has been shown that collective commitment to framing a conceptual model promotes collaboration sustainability [26,27]. This model framing process calls for dedication and readiness from all partners to incorporate their expertise into a mutually accepted all-encompassing model [47,49]. Participants in the consensus building process of a project ultimately “have a stake in the success of the project and are more likely to become involved in project design and execution” [49]. True partnerships also rely on consistent bi-directional learning [50]. Researchers should seek out and embrace local cultural structures, support community partners in cultivating their understanding of research methodologies, and be receptive to learning about and consciously engaging words or actions to communicate productively with and about the community [49,51]. Concurrently, community members should familiarize themselves with the fundamentals of community-engaged research to empower their position as research partners to provide valued community perspectives to best shape the CBPR project [43,45]. These processes serve to build social capital, solidarity, and credence among all involved in the project [50]. 

CSCs and CABs continually demonstrate their ability to successfully sustain CBPR partnerships. A critical element of this involves including stakeholders important to the community at hand to counteract mistrust among marginalized communities and to increase their research participation [52]. As in FAITH!, community members witnessed peers holding active, consequential roles as CSC members alongside the academic partners. This was imperative to easing the apprehension of some members concerning CBPR and shaping their understanding of the purpose of FAITH! in their community. Similarly, Ceasar and colleagues investigated the utility of a culturally sensitive CBPR research recruitment model among AAs with integration of a CAB into the project [5]. CAB recommendations garnered community trust and accordingly resulted in a more community-informed study design and intervention. Their CAB and the FAITH! CSC further substantiate the practice of incorporating such groups into CBPR, especially within understudied and under-resourced AA communities. They exemplify how devoted community members and academic researchers can partner to develop effective health interventions for the ultimate benefit of their communities [47].

## 5. Conclusions

Community members were successfully recruited to establish a robust and productive CSC to support an overarching CBPR project focused on CVH promotion in the AA community. Formation of the CSC was time-intensive and required integration of a strategic membership selection process and specific outline of CSC objectives. However, this process was essential to fostering genuine, inclusive, and productive relationships between the academic study team and community members. The FAITH! CSC has been vital to the growth of the academic-community partnership by ensuring its continued relevance and benefit to AA communities in MN. 

## Figures and Tables

**Table 1:** FAITH! Community Steering Committee Member Organizations.

FAITH! Community Steering Committee Member Organizations	
Community Organizations	Appetite for Change, Minneapolis, MN
	Hue-MAN Partnership, Minneapolis, MN
	Samuel Simmons Consulting, Minneapolis, MN
National/Regional Organizations	YMCA of the Greater Twin Cities, Minneapolis, MN
	American Heart Association, Minnesota Division
	Volunteers of America, Minnesota and Wisconsin Division
	Thrivent Financial, Rochester, MN
Faith-Based Organizations	Full Proof Ministry Church of God in Christ, Crystal, MN
	Christ’s Church of the Jesus Hour, Rochester MN
Academic Partners	Mayo Clinic Department of Cardiovascular Medicine, Rochester, MN
Health Systems	Mayo Clinic, Rochester, MN
	Allina Health, Minneapolis, MN
	Hennepin County Medical Center, Minneapolis, MN
Governmental Health Agencies	Minnesota Department of Health, St. Paul, MN
Health Insurance Organizations	BlueCross and BlueShield Minnesota, Eagan, MN

**Table 2:** FAITH! Community Steering Committee meeting evaluations: summary of responses by meeting.

	(1) Engagement Phase: Retreat (N = 8)	(2) Engagement Phase: Operating Procedures and Maintenance Meeting (N = 7)	(3) Covenant Development/ Empowerment Phase: Meeting 1 (N = 6)	(4) Covenant Development/ Empowerment Phase: Meeting 3 (N = 4)	Total (N = 25)
**I was notified of this meeting with sufficient notice**					
Missing	0	0	2	0	2
True	8 (100.0%)	7 (100.0%)	4 (100.0%)	4 (100.0%)	23 (100.0%)
**The meeting started and ended on time**					
Missing	1	2	2	0	5
True	7 (100.0%)	4 (80.0%)	4 (100.0%)	4 (100.0%)	19 (95.0%)
False	0 (0.0%)	1 (20.0%)	0 (0.0%)	0 (0.0%)	1 (5.0%)
**The meeting was worth my time**					
Missing	0	2	2	0	4
True	8 (100.0%)	4 (80.0%)	4 (100.0%)	4 (100.0%)	20 (95.2%)
Don’t know	0 (0.0%)	1 (20.0%)	0 (0.0%)	0 (0.0%)	1 (4.8%)
**Agenda**					
Good	3 (37.5%)	4 (57.1%)	3 (50.0%)	0 (0.0%)	10 (40.0%)
Excellent	5 (62.5%)	3 (42.9%)	3 (50.0%)	4 (100.0%)	15 (60.0%)
**The meeting objectives were accomplished**					
Missing	0	1	0	0	1
Fair	0 (0.0%)	2 (33.3%)	0 (0.0%)	0 (0.0%)	2 (8.3%)
Good	4 (50.0%)	1 (16.7%)	3 (50.0%)	0 (0.0%)	8 (33.3%)
Excellent	4 (50.0%)	3 (50.0%)	3 (50.0%)	4 (100.0%)	14 (58.3%)
**Location of meeting**					
Fair	0 (0.0%)	0 (0.0%)	1 (16.7%)	1 (25.0%)	2 (8.0%)
Good	2 (25.0%)	3 (42.9%)	4 (66.7%)	3 (75.0%)	12 (48.0%)
Excellent	6 (75.0%)	4 (57.1%)	1 (16.7%)	0 (0.0%)	11 (44.0%)
**Timing of meeting**					
Fair	1 (12.5%)	0 (0.0%)	0 (0.0%)	0 (0.0%)	1 (4.0%)
Good	3 (37.5%)	3 (42.9%)	3 (50.0%)	1 (25.0%)	10 (40.0%)
Excellent	4 (50.0%)	4 (57.1%)	3 (50.0%)	3 (75.0%)	14 (56.0%)
**Quality of facilitators/presenters**					
Good	1 (12.5%)	2 (28.6%)	2 (33.3%)	0 (0.0%)	5 (20.0%)
Excellent	7 (87.5%)	5 (71.4%)	4 (66.7%)	4 (100.0%)	20 (80.0%)
**Information shared at this meeting**					
Good	2 (25.0%)	4 (57.1%)	2 (33.3%)	0 (0.0%)	8 (32.0%)
Excellent	6 (75.0%)	3 (42.9%)	4 (66.7%)	4 (100.0%)	17 (68.0%)
**The handouts**					
Good	2 (25.0%)	3 (42.9%)	3 (50.0%)	0 (0.0%)	8 (32.0%)
Excellent	6 (75.0%)	4 (57.1%)	3 (50.0%)	4 (100.0%)	17 (68.0%)
**Opportunities for participation and sharing**					
Fair	0 (0.0%)	0 (0.0%)	1 (16.7%)	0 (0.0%)	1 (4.0%)
Good	2 (25.0%)	3 (42.9%)	2 (33.3%)	0 (0.0%)	7 (28.0%)
Excellent	6 (75.0%)	4 (57.1%)	3 (50.0%)	4 (100.0%)	17 (68.0%)
**The action plan or strategies developed**					
Missing	2	0	0	0	2
Fair	0 (0.0%)	3 (42.9%)	2 (33.3%)	0 (0.0%)	5 (21.7%)
Good	3 (50.0%)	3 (42.9%)	2 (33.3%)	0 (0.0%)	8 (34.8%)
Excellent	3 (50.0%)	1 (14.3%)	2 (33.3%)	4 (100.0%)	10 (43.5%)
**Outline of next steps** **/** **follow-up**					
Missing	2	2	0	0	4
Good	2 (33.3%)	3 (60.0%)	4 (66.7%)	0 (0.0%)	9 (42.9%)
Excellent	4 (66.7%)	2 (40.0%)	2 (33.3%)	4 (100.0%)	12 (57.1%)
**Food served**					
Good	2 (25.0%)	3 (42.9%)	4 (66.7%)	0 (0.0%)	9 (36.0%)
Excellent	6 (75.0%)	4 (57.1%)	2 (33.3%)	4 (100.0%)	16 (64.0%)

**Table 3:** 2018 FAITH! Community Steering Community (CSC) annual review member evaluations.

**Survey Results**	
	**Total** (n = 7)
**The CSC is fulfilling my expectations^1^**	
Agree	4 (57.1%)
Strongly agree	3 (42.9%)
**The CSC allows me to express my opinions and ideas^1^**	
Agree	1 (14.3%)
Strongly agree	6 (85.7%)
**The CSC has a clear mission/vision^1^**	
Agree	3 (42.9%)
Strongly agree	4 (57.1%)
**The CSC co-chairs are effective in leading the meetings^1^**	
Agree	5 (71.4%)
Strongly agree	2 (28.6%)
**The CSC co-chairs are effective in leading the project as a whole^1^**	
Neutral	1 (14.3%)
Agree	3 (42.9%)
Strongly Agree	3 (42.9%)
**CSC members are easy to work with^1^**	
Agree	3 (42.9%)
Strongly agree	4 (57.1%)
**The CSC FAITH! Covenant is clear and provides infrastructure to the group^1^**	
Neutral	1 (14.3%)
Agree	3 (42.9%)
Strongly agree	3 (42.9%)
**The number of CSC members is adequate^1^**	
Disagree	1 (14.3%)
Neutral	3 (42.9%)
Agree	2 (28.6%)
Strongly agree	1 (14.3%)
**The CSC has an action plan or strategies developed^1^**	
Neutral	1 (14.3%)
Agree	2 (28.6%)
Strongly agree	4 (57.1%)
**The CSC has an outline of next steps/follow up^1^**	
Neutral	1 (14.3%)
Agree	3 (42.9%)
Strongly agree	4 (57.1%)
**The CSC is focused on improving community health^1^**	
Strongly agree	7 (100.0%)
**Overall, how would you rate your satisfaction with being a CSC member? ^2^**	
Satisfied	4 (57.1%)
Very satisfied	3 (42.9%)
**Open-Ended Question Responses ^3^**	
**Is there anyone (or group representative) who should be a part of the CSC?**	
“It would be impactful for churches to commit time and staff to support and serve as advocates.”	
“Someone younger from a church group. Another voice outside the church leadership. Someone we are trying to serve/a participant.”	
**What amendments do you have to the FAITH! Covenant?**	
“Focus group participants – allowing input from a personal lens. Work more on using common language for community connections. Methods for achieving goals for personal and organizational perspectives.”	
**Is there anything that we did this year as a CSC that should be continued in the future?**	
“[Continue with] a future outline of meetings, projects, and the steps to prepare all presenting stuff as new knowledge for the team.”	
“…intermixing face to face meetings with conference calls is a good mix in managing time.”	
“Continue the communications about the project. Continue to think outside the box on community engagement.”	
“Continue to cross-check where our work connects with other similar work.”	
“Everything.”	
“[The] annual walk by FAITH!”	
**Do you have any other comments/feedback you would like to share?**	
“I believe in the mission and respect all the different expertise and opinions brought to the table.”	
“It would be good to hear more about the app and what progress is being made and how will it be introduced to address the needs of the health issues around cardiovascular health. Adding clarity of the work we are discussing at our meetings and how it is building the necessity feedback to create this service product for the community.”	

**^1^** Scale: Strongly Disagree to Strongly Agree; **^2^** Scale: Very Dissatisfied to Very Satisfied; **^3^** Displaying comments from individuals who did not respond with none/nothing at the moment.

## Appendix A. Sample Letter of Mutual Intent

### Letter of Mutual Intent for FAITH! (Fostering African-American Improvement in Total Health) Program Community Steering Committee

#### Introduction:

African-Americans in Minnesota are disproportionately affected by cardiovascular disease (CVD). Innovative and effective interventions developed with equitable engagement of communities most affected are essential towards improved CVD outcomes.

#### Desired Outcome:

To more effectively address the cardiovascular health disparities that exist in Minnesota and our urban, African-American faith communities through the ongoing efforts of the FAITH! (Fostering African-American Improvement in Total Health) Program.

#### Objectives:

To establish a community steering committee (CSC), specific to the FAITH! Program, composed of representatives from the local African-American faith community and community-based organizations to provide an infrastructure for community members to voice concerns, priorities and preferences in the development of a culturally-appropriate research intervention and process.

To define a process for formation, operation and maintenance of our academic–community partnership and CSC using a community-based participatory research (CBPR) approach by developing a FAITH! Covenant.

#### Specific Activities and Expectations:

Following formal agreement of all CSC members, a series of activities including a retreat will be held towards building a cohesive and effective CSC. The retreat will provide an overview of the tenets of CBPR and the overall goals of the academic-community partnership. The subsequent meetings (at least four over 6- to 12-month period) will focus on defining the CSC operating procedures, process evaluation and sustainability in the form of a FAITH! Covenant bylaws document as agreed upon and developed by the CSC. The frequency and timing of meetings will be determined by the CSC.

#### Summary

As a trusted community advocate, your participation as a member of this CSC will help enrich our efforts in developing culturally-appropriate research interventions for African-Americans and provide additional resources to local African-American faith communities. Your dedication to community engagement will enhance our credibility and capacity to foster improved total health for African-Americans. By signing this letter of mutual intent, we jointly agree that you will participate fully as a member of the FAITH! CSC as we work together to accomplish our desired outcomes and objectives.

#### ***Community Steering Committee*** ***Member***

_____________________________________

**Witnesses**

________________________________          _________________________________

**LaPrincess C. Brewer, MD, MPH**           **Clarence Jones, MEd**

FAITH! Program Academic Partner          FAITH! Program Community Partner

### Appendix B. FAITH! Community Steering Committee (CSC) Retreat Agenda

**Time**	**Agenda**
2:00–2:10 pm	**Welcome and Introductions**
2:10–2:15 pm	**Retreat Purpose**
	1. *To allow CSC members to engage with one another in a low-key environment to foster social interaction*
	2. *To build a cohesive and effective CSC*
	3. *To review the members’ role on the CSC and clarify expectations of the overarching FAITH! program partnership*
2:15–2:45 pm	**Group Discussion: Why I joined the FAITH! CSC**
2:45–2:55 pm	**Overview of Community-Based Participatory Research (CBPR) Key Principles**
	1. *Recognizes community as a unit of identity*
	2. *Builds on strengths and resources within the community*
	3. *Facilitates collaborative, equitable involvement of all partners in all phases of the research*
	4. *CBPR integrates knowledge and intervention for mutual benefit of all partners*
	5. *CBPR promotes a co-learning and empowering process that attends to social inequalities*
	6. *Involves a cyclical and iterative process*
	7. *Addresses health from both positive and ecological perspectives*
	8. *Disseminates findings and knowledge gained to all partners*
	9. *CBPR involves long-term commitment by all partners*
2:55–3:30 pm	**FAITH! Project background**
	Community-medical center partnership with African-American congregations and Mayo Clinic
	**Goals:**
	1. *Increase awareness of importance of healthy lifestyle in the prevention of heart disease in the African-American faith community*
	2. *Provide knowledge and tools on heart health to support health lifestyle*
	3. *Improve heart health in African-Americans*
3:30–3:50 pm	**Active Break**
3:50–4:05 pm	**Breakout Session 1: Elements of a successful community meeting**
	*List 5 elements of a successful and 5 elements of an unsuccessful meeting*
4:05–4:20 pm	**Breakout Session 2: Case story on trust-building**
	- *Read through the case story on “Group Trust in Flint/Lansing MI”*
	- *How can we build trust among each other within the CSC and with the FAITH! academic-community partners*
4:20–4:40 pm	**Questions and Answers**
4:40–5:00 pm	**Closing Remarks and Next Steps**
	- *Continue to reflect on your role and potential contributions as a FAITH! CSC member*
	- *Stay committed to upcoming CSC activities (Engagement, Covenant development and evaluation phases)*
